# Supplementary material for: Genome-Wide Identification and Expression Analysis of the PHD Finger Gene Family in Pea (Pisum sativum)
Source: Plants (Basel). 2024 May 28;13(11):1489. doi: 10.3390/plants13111489 (PMC11174613; doi:10.3390/plants13111489)
Supplement: Supplementary file 1 [file plants-13-01489-s001.zip › plants-2978409-supplementary.pdf]

Table S1

Basic information of *PsPHDs*

Gene name	Gene ID	Length (aa)	Molecular Weight （Da）	pI	Instability Index	Aliphatic Index	Hydropathicity	subcellular localization
PsPHD1	Psat01G0035100	1232	136687.6	6.54	48.44	86.6	-0.295	Chloroplast. Nucleus.
PsPHD2	Psat01G0103300	644	71491.8	5.82	54.67	78.09	-0.465	Nucleus
PsPHD3	Psat01G0150300	2226	245193	5.35	44.49	80.25	-0.416	Nucleus
PsPHD4	Psat01G0172400	1801	202149.6	6.16	45.02	97.13	-0.164	Nucleus
PsPHD5	Psat01G0250600	1241	139648.3	6.8	62.51	63.19	-0.882	Nucleus
PsPHD6	Psat01G0322300	755	83978.06	7.52	37.79	84.87	-0.191	Nucleus
PsPHD7	Psat01G0367400	265	30108.92	4.45	82.92	56.34	-0.892	Nucleus
PsPHD8	Psat01G0398100	724	82393.31	8.88	45.42	64.64	-1.115	Nucleus
PsPHD9	Psat02G0009200	565	64037.89	6.43	52.55	78.78	-0.543	Nucleus
PsPHD10	Psat02G0026700	821	88835.77	5.76	51.37	72.86	-0.432	Nucleus
PsPHD11	Psat02G0148300	744	84143.78	6.24	47.03	79.77	-0.319	Nucleus
PsPHD12	Psat02G0182600	1126	129142.9	6.63	47.87	78.45	-0.567	Nucleus
PsPHD13	Psat02G0198800	1682	190492.6	6.15	50.93	84.58	-0.419	Nucleus
PsPHD14	Psat02G0198900	1682	190492.6	6.15	50.93	84.58	-0.419	Nucleus
PsPHD15	Psat02G0206300	872	98859.9	8.25	40.41	82.67	-0.192	Nucleus
PsPHD16	Psat02G0352600	660	75348.08	7.09	46.16	91.5	-0.185	Nucleus
PsPHD17	Psat02G0534800	1826	208151.7	7.98	48.34	88.66	-0.312	Nucleus
PsPHD18	Psat02G0553900	1544	170131.9	5.55	52.04	71.17	-0.758	Nucleus
PsPHD19	Psat03G0014500	1049	117797	8.67	42.6	72.56	-0.45	Nucleus
PsPHD20	Psat03G0014600	1030	115420.7	8.34	41.47	71.92	-0.49	Nucleus
PsPHD21	Psat03G0061900	924	105135.8	4.97	79.02	58.1	-1.019	Nucleus
PsPHD22	Psat03G0151300	1188	130278.2	9.21	44.38	77.19	-0.349	Nucleus

PsPHD23	Psat03G0162700	1493	169471.8	7.14	44.13	83.73	-0.465	Nucleus
PsPHD24	Psat03G0200400	193	21591.29	4.85	38.87	62.69	-0.362	Nucleus
PsPHD25	Psat03G0218900	312	35916.53	7.09	57.06	76.54	-0.158	Nucleus
PsPHD26	Psat03G0417700	241	27203.49	5.3	58.31	67.59	-0.661	Nucleus
PsPHD27	Psat03G0573800	1098	123376.1	7.7	41.67	75.99	-0.467	Nucleus
PsPHD28	Psat04G0003200	260	29208.74	5.01	48.08	59.31	-0.715	Nucleus
PsPHD29	Psat04G0044200	727	79954.25	5.7	52.26	74.68	-0.539	Nucleus
PsPHD30	Psat04G0152700	617	68989.68	8.61	50.56	65.36	-0.67	Nucleus
PsPHD31	Psat04G0420100	218	25024.19	7.52	46.44	56.28	-0.699	Nucleus
PsPHD32	Psat04G0472600	1316	145399.4	6.43	54.24	84.4	-0.328	Chloroplast. Nucleus
PsPHD33	Psat04G0546100	1023	116110.8	8.48	42.87	68.66	-0.512	Nucleus
PsPHD34	Psat04G0578000	303	34792.05	5.59	46.99	62.44	-0.785	Nucleus
PsPHD35	Psat04G0642600	529	58778.71	8.92	47.87	81.15	-0.376	Nucleus
PsPHD36	Psat05G0014200	218	24964.16	7.56	47.7	53.21	-0.716	Nucleus
PsPHD37	Psat05G0110900	1427	163346.8	5.39	42.28	80.15	-0.632	Nucleus
PsPHD38	Psat05G0135500	627	71129.62	7.76	48.9	75.34	-0.319	Nucleus
PsPHD39	Psat05G0158700	495	55455.85	5.75	49.36	74.81	-0.53	Nucleus
PsPHD40	Psat05G0197300	412	47197.41	9.58	51.56	68.64	-0.584	Nucleus
PsPHD41	Psat05G0239900	1062	120573.9	8.79	42.29	71.21	-0.506	Nucleus
PsPHD42	Psat05G0256000	877	95678.36	5.16	39.93	65.3	-0.557	Nucleus
PsPHD43	Psat05G0393700	1482	163947.5	6.79	48.2	76.7	-0.458	Nucleus
PsPHD44	Psat05G0448700	915	103857	8.67	56.99	78.44	-0.405	Nucleus
PsPHD45	Psat05G0670400	1321	146137.4	6.3	43.65	77.15	-0.407	Nucleus
PsPHD46	Psat05G0673000	218	24865.24	8.59	45.37	49.59	-0.837	Nucleus
PsPHD47	Psat05G0712400	1061	116603.1	5.44	35.69	82.74	-0.31	Nucleus
PsPHD48	Psat05G0714700	1720	192805.2	6.21	52.72	77.3	-0.463	Nucleus

PsPHD49	Psat05G0752400	192	22381.72	8.75	43.58	60.89	-0.536	Nucleus
PsPHD50	Psat05G0767200	913	99489.72	5.05	37.44	81.3	-0.382	Nucleus
PsPHD51	Psat05G0801300	249	28292.86	5.17	48.18	66.59	-0.686	Nucleus
PsPHD52	Psat06G0062300	268	30355.92	4.75	49.2	59.7	-0.691	Nucleus
PsPHD53	Psat06G0096800	1042	117880.4	9.04	35.92	73.08	-0.479	Nucleus
PsPHD54	Psat06G0099900	348	39103.91	8.96	56.93	73.94	-0.466	Nucleus
PsPHD55	Psat06G0143300	1186	131013.1	8.64	47.66	54.23	-1.027	Nucleus
PsPHD56	Psat06G0168000	1574	172862.6	5.7	44.47	75.83	-0.619	Nucleus
PsPHD57	Psat06G0183400	880	98167.79	5.84	40.98	78.53	-0.451	Nucleus
PsPHD58	Psat06G0207800	831	93296.19	9.14	51	84.48	-0.313	Nucleus
PsPHD59	Psat06G0262200	347	39190.14	5.95	48.25	73.63	-0.507	Nucleus
PsPHD60	Psat06G0443700	1353	148389.7	7.63	37.62	70.43	-0.675	Nucleus
PsPHD61	Psat06G0508500	466	51967.53	5.23	45.72	78.71	-0.521	Nucleus
PsPHD62	Psat06G0560700	466	51644.27	5.34	45.49	76.44	-0.476	Nucleus
PsPHD63	Psat06G0560800	492	54824.77	5.49	45.84	72.2	-0.534	Nucleus
PsPHD64	Psat06G0562700	1591	174664.2	5.92	44.54	70.12	-0.716	Nucleus
PsPHD65	Psat06G0582400	1313	141595.1	4.94	48.98	60.88	-1.004	Nucleus
PsPHD66	Psat06G0588000	520	58284.18	5.19	56.58	83.62	-0.244	Nucleus
PsPHD67	Psat07G0037800	735	81476.39	7.97	56.36	74.16	-0.467	Nucleus
PsPHD68	Psat07G0080300	368	41868.21	9.29	33.5	85.24	-0.151	Nucleus
PsPHD69	Psat07G0209400	827	92133.28	5.94	43.95	67.41	-0.638	Nucleus
PsPHD70	Psat07G0228200	2321	258536	7.92	50.08	74.27	-0.695	Nucleus
PsPHD71	Psat07G0411400	1627	182001.6	8.47	48.93	79.48	-0.48	Nucleus
PsPHD72	Psat07G0450600	1693	188761.8	6.04	50.97	75.14	-0.427	Nucleus
PsPHD73	Psat07G0455100	704	78822.1	7.93	44.31	82.17	-0.173	Nucleus
PsPHD74	Psat07G0455300	704	78822.1	7.93	44.31	82.17	-0.173	Nucleus

PsPHD75	Psat07G0455500	715	80319.7	8.04	42.93	78.71	-0.25	Nucleus
PsPHD76	Psat07G0455600	802	89740.18	8.58	45.78	79.26	-0.342	Nucleus
PsPHD77	Psat07G0455800	568	63789.42	7.71	44.36	82.78	-0.284	Nucleus
PsPHD78	Psat07G0474000	695	78283.67	8.59	33.65	80.07	-0.259	Nucleus
PsPHD79	Psat07G0487200	862	96285.23	8.98	48.73	60.48	-0.798	Nucleus
PsPHD80	Psat07G0489100	1367	152408.6	5.96	56.78	63.65	-0.805	Nucleus
PsPHD81	Psat07G0610400	889	96525.29	5.02	38.34	80.36	-0.389	Nucleus
PsPHD82	Psat07G0670700	241	27274.58	5.29	55.17	67.14	-0.676	Nucleus
PsPHD83	Psat07G0688400	1692	191238.5	8.15	54.5	66.84	-0.685	Nucleus
PsPHD84	chrUn0247G0000200	682	76327.93	7.63	44.32	79.24	-0.277	Nucleus

Table S2
Basic information for the 20 subfamilies

Subfamily	ID	species type
G	AT1G77800.3	*Arabidopsis thaliana*
	AT3G05670.1	*Arabidopsis thaliana*
	PsPHD21	*Pisum sativum*
	MTR_7g112410	*Medicago truncatula*
	MTR_8g042510	*Medicago truncatula*
	AT3G14740.2	*Arabidopsis thaliana*
	AT1G68030.1	*Arabidopsis thaliana*
	AT3G19510.1	*Arabidopsis thaliana*
	MTR_1g107015	*Medicago truncatula*
K	AT5G12400.1	*Arabidopsis thaliana*
	MTR_4g076180	*Medicago truncatula*
	PsPHD71	*Pisum sativum*
	PsPHD23	*Pisum sativum*
	PsPHD4	*Pisum sativum*
	PsPHD72	*Pisum sativum*
	MTR_4g055920	*Medicago truncatula*
	AT5G35210.1	*Arabidopsis thaliana*
	AT5G22760.1	*Arabidopsis thaliana*
	PsPHD48	*Pisum sativum*
	MTR_2g084615	*Medicago truncatula*
	MTR_2g084620	*Medicago truncatula*
D	PsPHD55	*Pisum sativum*
	AT1G32810.2	*Arabidopsis thaliana*
	PsPHD27	*Pisum sativum*
	MTR_7g021365	*Medicago truncatula*
	AT1G05830.1	*Arabidopsis thaliana*
	AT2G31650.1	*Arabidopsis thaliana*
	PsPHD68	*Pisum sativum*
	AT4G27910.1	*Arabidopsis thaliana*
	AT5G53430.1	*Arabidopsis thaliana*
	PsPHD53	*Pisum sativum*
	MTR_1g008230	*Medicago truncatula*
	MTR_3g091310	*Medicago truncatula*
	PsPHD41	*Pisum sativum*
	AT3G61740.1	*Arabidopsis thaliana*

	PsPHD15	*Pisum sativum*
	PsPHD5	*Pisum sativum*
	AT5G60410.2	*Arabidopsis thaliana*
	PsPHD50	*Pisum sativum*
	PsPHD81	*Pisum sativum*
	MTR_4g060510	*Medicago truncatula*
	AT2G25170.3	*Arabidopsis thaliana*
	PsPHD37	*Pisum sativum*
	MTR_3g106210	*Medicago truncatula*
	AT5G44800.1	*Arabidopsis thaliana*
	PsPHD70	*Pisum sativum*
	MTR_4g096930	*Medicago truncatula*
S	MTR_2g038000	*Medicago truncatula*
	AT4G29940.1	*Arabidopsis thaliana*
	PsPHD8	*Pisum sativum*
	AT2G19260.1	*Arabidopsis thaliana*
	PsPHD66	*Pisum sativum*
	PsPHD39	*Pisum sativum*
	MTR_3g100350	*Medicago truncatula*
I	AT4G10600.1	*Arabidopsis thaliana*
	MTR_8g447090	*Medicago truncatula*
	PsPHD32	*Pisum sativum*
	AT1G79350.1	*Arabidopsis thaliana*
	PsPHD1	*Pisum sativum*
	MTR_2g063620	*Medicago truncatula*
O	PsPHD25	*Pisum sativum*
	MTR_7g095140	*Medicago truncatula*
	PsPHD45	*Pisum sativum*
	AT3G14980.1	*Arabidopsis thaliana*
	MTR_2g078040	*Medicago truncatula*
	PsPHD56	*Pisum sativum*
	AT5G36670.1	*Arabidopsis thaliana*
	AT5G36740.1	*Arabidopsis thaliana*
	AT1G05380.1	*Arabidopsis thaliana*
	PsPHD64	*Pisum sativum*
	MTR_1g103830	*Medicago truncatula*
	AT1G63490.4	*Arabidopsis thaliana*
	PsPHD17	*Pisum sativum*
	MTR_5g010300	*Medicago truncatula*
	MTR_3g103160	*Medicago truncatula*
	PsPHD38	*Pisum sativum*
	MTR_3g103120	*Medicago truncatula*
	MTR_3g103110	*Medicago truncatula*
	MTR_7g109640	*Medicago truncatula*
	MTR_5g024390	*Medicago truncatula*
	AT5G58610.3	*Arabidopsis thaliana*
	AT5G63900.1	*Arabidopsis thaliana*
	MTR_4g099530	*Medicago truncatula*
	PsPHD12	*Pisum sativum*

	MTR_5g071340	*Medicago truncatula*
	PsPHD44	*Pisum sativum*
	MTR_3g464550	*Medicago truncatula*
M	PsPHD35	*Pisum sativum*
	PsPHD61	*Pisum sativum*
	PsPHD62	*Pisum sativum*
	PsPHD63	*Pisum sativum*
	AT5G22260.1	*Arabidopsis thaliana*
	PsPHD16	*Pisum sativum*
	MTR_5g040700	*Medicago truncatula*
	AT2G01810.1	*Arabidopsis thaliana*
	AT1G66170.1	*Arabidopsis thaliana*
	PsPHD11	*Pisum sativum*
	MTR_5g077750	*Medicago truncatula*
	PsPHD6	*Pisum sativum*
	MTR_1g076990	*Medicago truncatula*
	MTR_4g035550	*Medicago truncatula*
	AT1G33420.1	*Arabidopsis thaliana*
	PsPHD78	*Pisum sativum*
R	PsPHD47	*Pisum sativum*
	PsPHD77	*Pisum sativum*
	PsPHD73	*Pisum sativum*
	PsPHD84	*Pisum sativum*
	PsPHD75	*Pisum sativum*
	PsPHD76	*Pisum sativum*
	PsPHD74	*Pisum sativum*
	AT2G27980.1	*Arabidopsis thaliana*
	AT2G36720.1	*Arabidopsis thaliana*
	PsPHD58	*Pisum sativum*
	PsPHD22	*Pisum sativum*
	MTR_7g102610	*Medicago truncatula*
	AT3G53680.2	*Arabidopsis thaliana*
	AT2G37520.1	*Arabidopsis thaliana*
	AT1G14770.1	*Arabidopsis thaliana*
	MTR_1g068930	*Medicago truncatula*
	PsPHD24	*Pisum sativum*
	PsPHD57	*Pisum sativum*
E	PsPHD80	*Pisum sativum*
	MTR_3g088665	*Medicago truncatula*
	PsPHD42	*Pisum sativum*
	PsPHD65	*Pisum sativum*
	AT1G77250.1	*Arabidopsis thaliana*
	MTR_8g099585	*Medicago truncatula*
	MTR_1g007700	*Medicago truncatula*
	AT1G50620.1	*Arabidopsis thaliana*
	PsPHD10	*Pisum sativum*
	MTR_5g093940	*Medicago truncatula*
T	AT5G15540.1	*Arabidopsis thaliana*
	MTR_1g012910	*Medicago truncatula*

	AT3G01460.1	*Arabidopsis thaliana*
	PsPHD3	*Pisum sativum*
	MTR_6g007650	*Medicago truncatula*
Q	MTR_2g090365	*Medicago truncatula*
	MTR_3g117270	*Medicago truncatula*
	MTR_3g117280	*Medicago truncatula*
	PsPHD36	*Pisum sativum*
	PsPHD49	*Pisum sativum*
	AT4G39100.1	*Arabidopsis thaliana*
	AT4G22140.1	*Arabidopsis thaliana*
	PsPHD31	*Pisum sativum*
	PsPHD46	*Pisum sativum*
	MTR_2g078310	*Medicago truncatula*
J	MTR_1g087290	*Medicago truncatula*
	PsPHD43	*Pisum sativum*
	MTR_3g073320	*Medicago truncatula*
	AT1G54390.2	*Arabidopsis thaliana*
	MTR_7g085450	*Medicago truncatula*
P	MTR_5g067980	*Medicago truncatula*
	PsPHD13	*Pisum sativum*
	PsPHD14	*Pisum sativum*
	AT3G20280.1	*Arabidopsis thaliana*
	AT1G43770.2	*Arabidopsis thaliana*
	PsPHD59	*Pisum sativum*
	AT4G14700.1	*Arabidopsis thaliana*
	MTR_8g099640	*Medicago truncatula*
	AT4G12620.1	*Arabidopsis thaliana*
A	AT1G57820.1	*Arabidopsis thaliana*
	AT1G57800.2	*Arabidopsis thaliana*
	AT1G66050.1	*Arabidopsis thaliana*
	AT5G39550.1	*Arabidopsis thaliana*
	AT1G66040.1	*Arabidopsis thaliana*
F	PsPHD30	*Pisum sativum*
	MTR_4g110090	*Medicago truncatula*
	AT5G57380.1	*Arabidopsis thaliana*
	PsPHD29	*Pisum sativum*
	PsPHD2	*Pisum sativum*
	PsPHD67	*Pisum sativum*
	AT3G52100.1	*Arabidopsis thaliana*
	AT3G08020.1	*Arabidopsis thaliana*
	PsPHD79	*Pisum sativum*
	MTR_4g037745	*Medicago truncatula*
C	AT5G16680.1	*Arabidopsis thaliana*
	AT3G02890.1	*Arabidopsis thaliana*
	PsPHD18	*Pisum sativum*
	MTR_0001s0570	*Medicago truncatula*
B	PsPHD40	*Pisum sativum*
	MTR_3g095840	*Medicago truncatula*
	AT5G24330.1	*Arabidopsis thaliana*

	AT5G09790.2	*Arabidopsis thaliana*
	PsPHD54	*Pisum sativum*
	MTR_1g007670	*Medicago truncatula*
L	PsPHD28	*Pisum sativum*
	MTR_4g134510	*Medicago truncatula*
	AT1G14510.1	*Arabidopsis thaliana*
	AT2G02470.1	*Arabidopsis thaliana*
	AT3G42790.1	*Arabidopsis thaliana*
	AT3G11200.1	*Arabidopsis thaliana*
	AT5G05610.1	*Arabidopsis thaliana*
	PsPHD82	*Pisum sativum*
	MTR_4g015830	*Medicago truncatula*
	MTR_7g068590	*Medicago truncatula*
	PsPHD26	*Pisum sativum*
	PsPHD52	*Pisum sativum*
	MTR_1g015185	*Medicago truncatula*
	AT3G24010.1	*Arabidopsis thaliana*
	AT5G26210.1	*Arabidopsis thaliana*
	AT5G20510.1	*Arabidopsis thaliana*
	PsPHD7	*Pisum sativum*
	MTR_2g040990	*Medicago truncatula*
	PsPHD34	*Pisum sativum*
	MTR_8g019520	*Medicago truncatula*
	PsPHD51	*Pisum sativum*
	MTR_2g099500	*Medicago truncatula*

Table S3
Detailed information on cis-acting element analysis of the PHD finger family genes in pea.

function	Element type	Sequence	Length	Number	function
stress response	ARE	AAACCA	6	145	anaerobic induction
	GC-motif	CCCCCG	6	6	anoxic specific inducibility
	TC-rich repeats	ATTCTCTAAC	9	51	defense and stress responsiveness
	MBS	CAACTG	6	53	drought-inducibility
	MBSI	TTTTTACGGTTA	11	13	flavonoid biosynthetic genes regulation
	LTR	CCGAAA	6	55	low-temperature responsiveness
	AT-rich sequence	TAAAATACT	9	18	maximal elicitor-mediated activation
	WUN-motif	AAATTTCTT	9	63	wound-responsive element
plant hormone response	ABRE	ACGTG	5	148	abscisic acid responsiveness
	TGA-element	AACGAC	6	37	auxin responsiveness
	AuxRR-core	GGTCCAT	7	7	
	TGA-box	TGACGTAA	8	4	
	AuxRE	TGTCTCAATAAG	11	2	
	TATC-box	TATCCCA	7	21	gibberellin-responsiveness
	GARE-motif	TCTGTTG	7	17	
	P-box	CCTTTTG	7	17	
	CGTCA-motif	CGTCA	5	121	MeJA-responsiveness
	TGACG-motif	TGACG	5	121	
	TCA-element	CCATCTTTTT	9	45	salicylic acid responsiveness
	SARE	TTCGACCATCTT	11	1	
plant growth and development	MSA-like	TCAAACGGT	9	4	cell cycle regulation
	HD-Zip 1	CAAT(A/T)ATTG	8	5	differentiation of the palisade mesophyll cells
	GCN4_motif	TGAGTCA	7	20	endosperm expression
	AACA_motif	TAACAAACTCCA	11	1	
	CAT-box	GCCACT	6	26	meristem expression
	NON-box	AGATCGACG	9	1	
	RY-element	CATGCATG	8	5	seed-specific regulation
	O2-site	GTTGACGTGA	10	45	zein metabolism regulation
	Box 4	ATTAAT	6	325	light responsiveness
	G-Box	CACGTT	6	174	
	GT1-motif	GGTTAA	6	91	

	GATA-motif	GATAGGG	7	76	
	TCT-motif	TCTTAC	6	67	
	I-box	TGATAATGT	9	47	
	MRE	AACCTAA	7	47	
	AE-box	AGAAACTT	8	41	
	ATCT-motif	AATCTAATCC	9	19	
	AT1-motif	AATTATTTTTATT	13	18	
	TCCC-motif	TCTCCCT	7	16	
	ACE	CTAACGTATT	9	15	
	GA-motif	ATAGATAA	8	15	
	chs-CMA1a	TTACTTAA	8	12	
	Gap-box	CAAATGAA(A/G)A	9	11	
	ATC-motif	AGTAATCT	8	9	
	LAMP-element	CTTTATCA	8	8	
	chs-CMA2a	TCACTTGA	8	7	
	3-AF1 binding site	TAAGAGAGGAA	10	6	
	Sp1	GGGCGG	6	5	
	Box II	TGGTAATAA	9	2	
	CAG-motif	GAAAGGCAGAC	10	2	
	GTGGC-motif	CATCGTGTGGC	10	2	
	GA-motif	ATAGATAA	8	15	
	chs-CMA1a	TTACTTAA	8	12	
	Gap-box	CAAATGAA(A/G)A	9	11	
	ATC-motif	AGTAATCT	8	9	
	LAMP-element	CTTTATCA	8	8	
	chs-CMA2a	TCACTTGA	8	7	
	3-AF1 binding site	TAAGAGAGGAA	10	6	
	Sp1	GGGCGG	6	5	
	Box II	TGGTAATAA	9	2	
	CAG-motif	GAAAGGCAGAC	10	2	
	GTGGC-motif	CATCGTGTGGC	10	2	
	AAAC-motif	CAATCAAAACCT	11	1	
	ACA-motif	AATTACAGCCATT	12	1	
	GATT-motif	CTCCTGATTAGC	11	1	
	LS7	CAGATTTATTTTA	13	1	
	sbp-CMA1c	CTTTATCTCTTCCA	13	1	
	AT-rich	ATAGAAATCAA	10	9	binding site of

	element				AT-rich DNA binding protein (ATBP-1)
	circadian	CAAAGATATC	9	16	circadian control
	CAAT-box	CAAAT	5	3402	common cis-acting element in promoter and enhancer regions
	TATA-box	TATAAA	6	6417	core promoter element around -30 of transcription start
	3-AF3 binding site	CACTATCTAAC	10	2	DNA module array (CMA3)
	CCAAT-box	CAACGG	6	27	MYBHv1 binding site
	Box III	atCATTTTCACt	11	5	protein binding site
	HD-Zip 3	GTAAT(G/C)ATTAC	9.5	1	

Table S4
Statistics of cis-acting elements in several genes of the PHD finger family in pea.

**cis-acting elements**	**PsP HD6**	**PsPH D11**	**PsPH D16**	**PsPH D35**	**PsPH D61**	**PsPH D62**	**PsPH D63**	**PsPH D78**
anaerobic induction	2	0	4	1	4	0	0	2
acid responsiveness	0	1	0	1	0	0	2	0
defense and stress responsiveness	0	0	1	0	1	0	0	1
low-temperature responsiveness	1	0	0	1	0	0	0	0
wound-responsive	0	0	0	0	0	0	0	1
drought-inducibility	0	1	1	0	1	0	0	0
flavonoid biosynthetic genes regulation	0	0	0	0	0	0	1	0
MeJA-responsiveness	4	4	2	4	0	4	6	2
auxin-responsiveness	1	0	0	0	0	1	2	2
abscisic acid responsiveness	0	1	0	0	0	0	1	0
gibberellin-responsiv eness	0	2	1	0	1	0	0	0
light responsiveness	23	14	4	19	8	14	16	17
meristem expression	0	1	1	0	0	0	1	1
seed-specific regulation	0	0	0	0	0	0	1	0
circadian control	0	0	1	0	2	1	0	0
endosperm expression	0	1	1	0	0	0	0	0
zein metabolism regulation	0	0	1	0	0	0	0	0
protein binding site	0	0	0	0	0	1	0	0
binding site of AT-rich DNA binding protein (ATBP-1)	0	0	0	0	0	0	0	1
MYBHv1 binding site	0	0	0	0	1	0	2	0

Table S5

The RT-qPCR primers for PsPHDs.

Primer name	Forward/reverse	sequence(5'→3')
PsPHD35	F	CATCTGGAATGTGCGCTTCG
	R	CAGACAAGCGCCAAGGTCTA
PsPHD16	F	ACGAGGGTTATCGTTGAGGC
	R	AGCTCCGGCGAACCAAATAG
PsPHD11	F	GAGGCGGTGGAATTAGCAGT
	R	CAAGCTCACCACAAGTTGGC
PsPHD6	F	CTCCACCGTCAGATCTCCAAC
	R	CGGAGAAGTTGCCTTCCGAC
PsPHD78	F	TCTGGGATAGGCTCTGTGCT
	R	GGACAGCGTATCAACTGCCT
PsEF1a	F	GATGCACCTGGACATCGTGAC
	R	CTTAGGGGTGGTAGCATCCATCT
